# Prognostic Biomarkers for Esophageal Adenocarcinoma Identified by Analysis of Tumor Transcriptome

**DOI:** 10.1371/journal.pone.0015074

**Published:** 2010-11-30

**Authors:** Soo Mi Kim, Yun-Yong Park, Eun Sung Park, Jae Yong Cho, Julie G. Izzo, Di Zhang, Sang-Bae Kim, Jeffrey H. Lee, Manoop S. Bhutani, Stephen G. Swisher, Xifeng Wu, Kevin R. Coombes, Dipen Maru, Kenneth K. Wang, Navtej S. Buttar, Jaffer A. Ajani, Ju-Seog Lee

**Affiliations:** 1 Department of Systems Biology, University of Texas MD Anderson Cancer Center, Houston, Texas, United States of America; 2 Department of Experimental Therapeutics, University of Texas MD Anderson Cancer Center, Houston, Texas, United States of America; 3 Department of Epidemiology, University of Texas MD Anderson Cancer Center, Houston, Texas, United States of America; 4 Department of Gastrointestinal Medical Oncology, University of Texas MD Anderson Cancer Center, Houston, Texas, United States of America; 5 Department of Bioinformatics and Computational Biology, University of Texas MD Anderson Cancer Center, Houston, Texas, United States of America; 6 Department of Pathology, University of Texas MD Anderson Cancer Center, Houston, Texas, United States of America; 7 Barrett Esophagus Unit, Division of Gastroenterology and Hepatology, Mayo Clinic College of Medicine, Rochester, Minnesota, United States of America; 8 Department of Physiology, Chonbuk National University Medical School and Hospital, Jeonju, Korea; Duke-NUS Graduate Medical School, Singapore

## Abstract

**Background:**

Despite many attempts to establish pre-treatment prognostic markers to understand the clinical biology of esophageal adenocarcinoma (EAC), validated clinical biomarkers or parameters remain elusive. We generated and analyzed tumor transcriptome to develop a practical biomarker prognostic signature in EAC.

**Methodology/Principal Findings:**

Untreated esophageal endoscopic biopsy specimens were obtained from 64 patients undergoing surgery and chemoradiation. Using DNA microarray technology, genome-wide gene expression profiling was performed on 75 untreated cancer specimens from 64 EAC patients. By applying various statistical and informatical methods to gene expression data, we discovered distinct subgroups of EAC with differences in overall gene expression patterns and identified potential biomarkers significantly associated with prognosis. The candidate marker genes were further explored in formalin-fixed, paraffin-embedded tissues from an independent cohort (52 patients) using quantitative RT-PCR to measure gene expression. We identified two genes whose expression was associated with overall survival in 52 EAC patients and the combined 2-gene expression signature was independently associated with poor outcome (*P*<0.024) in the multivariate Cox hazard regression analysis.

**Conclusions/Significance:**

Our findings suggest that the molecular gene expression signatures are associated with prognosis of EAC patients and can be assessed prior to any therapy. This signature could provide important improvement for the management of EAC patients.

## Introduction

Esophageal adenocarcinoma (EAC) is one of high mortality cancers in the West and having a 5-year survival rate of less than 10% [1], [2]. The occurrence of EAC has increased over the past 20 years [3]. In the United States, it was projected that there were more than 14,000 deaths from EAC in 2009 [4]. The reason for this increase in EAC is unknown. Several studies have shown that at least 95% of EAC cases arise from the metaplastic condition known as Barrett's esophagus, which is caused by gastroesophageal reflux disease [5], [6]. Surgery is the best curative treatment option but only a small fraction of EAC patients benefit because many patients still suffer from recurrence within 2 years after curative treatment [2], [7]. Despite continual efforts to preoperatively select patients who are likely to benefit from potentially curative surgery, the current staging system, which uses TNM stage and lymph node status, has shown limited success in predicting the duration of overall survival (OS) or recurrence-free survival (RFS) in EAC patients [8].

Gene expression profiling studies of various cancers have discovered consistent gene expression patterns associated with pathological or clinical phenotype, elucidating subtypes of cancer previously unidentified with conventional technologies [9]–[13]. Therefore, we investigated the possibility that gene-expression variations found in EAC biopsy samples, obtained prior to administering any therapy, would permit the identification of distinct subclasses of EAC patients with different prognoses. Our goal was to identify a subgroup of patients who do not derive much benefit from combined modality therapy and have a very poor prognosis. Our results revealed three subclasses of EAC patients characterized by significant differences in gene expression that correlated with prognosis. We also identified expression profiles for a limited number of genes that accurately predicted prognosis and explored the possibility for the use of the expression signature as prognostic marker.

## Methods

### Participants and Ethics

One-hundred-and-sixteen EAC patients were included in the study. Esophageal specimens were obtained from patients undergoing esophagectomy as primary treatment of EAC at The University of Texas M. D. Anderson Cancer Center (MDACC). Pathologic staging was done according to the criteria of the American Joint Committee on Cancer [14]. Histological confirmation of the diagnosis was established in all patients. Postsurgical surveillance was done every three months during the first year. Thereafter, it was done every six months for two additional years, and then yearly. Five to 6 weeks after the completion of chemoradiation, all patients underwent resection of the esophagus and regional lymph nodes. The type of surgery was determined by the location of the primary tumor, condition of the patient, and surgeon's preference. The commonly performed procedure was Ivor-Lewis esophagogastrectomy.

Seventy-five frozen biopsy specimens of tumors and 28 paired surrounding non-tumor esophageal (NE) tissues endoscopically obtained before treatment from 2002 through 2007 from 64 EAC patients were selected from fresh-frozen tissue bank of The University of Texas M. D. Anderson Cancer Center for microarray experiments. Furthermore, 15 Barrett's esophagus frozen biopsy specimens from Mayo Clinic were included as pre-cancerous tissue specimens. All samples were collected after obtaining written informed consent from patients and our study was approved by the Institutional Review Board (IRB) at the University of Texas MD Anderson Cancer Center. Clinical data were obtained retrospectively, and table 1 shows the characteristics of the patients with EAC. To validate the levels of gene expression found by microarray analysis, quantitative reverse transcription-polymerase chain reaction (qRT-PCR) experiments were performed with formalin-fixed, paraffin-embedded (FFPE) tissues from an independent EAC patient group (N = 52). Tissue specimens used in qRT-PCR were obtained retrospectively from the surgical specimens.

**Table 1:** Characteristics of patients and tissues.

Variable	Explorationcohort	Secondcohort	P value(χ^2^ test)	Total
**Gender**	64	52	0.98	**116**
** Male**	59	48		**107**
** Female**	5	4		**9**
**Race**			0.076	
** White**	58	52		**58**
** Latino**	5	0		**5**
** Asian**	1	0		**1**
**Age**			0.21*	
** Mean**	63.2	60.44		
** SE**	1.5	11.57		
**Barrett's** #			0.46	
** +**	20	14		**34**
** −**	40	38		**78**
**Smoking** †			0.002	
** +**	14	25		**39**
** −**	50	26		**76**
**Stage** ‡			0.15	
** I**	2	5		**7**
** II**	23	11		**34**
** III**	30	29		**59**
** IV**	5	7		**12**
**Relapse**				
	20	26		**46**
**Death**				
	21	44		**65**

*Student t-test.

# Four cases were not available.

† One case was not available.

‡ Four cases were not available.

### Isolation of RNA

Total RNA was extracted from the frozen tissues by using a mirVana^TM^ miRNA isolation labeling kit (Ambion Inc., TX, USA). The total RNA was quantified by using a Nanodrop ND-1000 spectrophotometer (NanoDrop Technology, DE, USA), and the integrity of the large RNA fraction was determined with an Experion^TM^ (BIO-RAD, CA, USA) as a surrogate for mRNA quality control. The total RNA samples with adequate RNA quality index (>7) were used for microarray analysis.

### Labeling and Hybridization of mRNA, Scanning, Data Processing, and Data Analysis

Five-hundred ng of total RNA was used for labeling and hybridization, according to the manufacturer's protocols (Illumina Inc., CA, USA). The hybridized biotinylated cRNA was detected with 1 µg/ml cyanine 3-streptavidine (GE Healthcare, NJ, USA), and the bead chips were scanned with an Illumina BeadArray Reader (Illumina, CA, USA). The microarray data were extracted with Bead Studio 3.6 (Illumina, CA, USA) and normalized using the quantile normalization method in the Linear Models for Microarray Data (LIMMA) package in R language environment [15]. The expression level of each gene was transformed into a log 2 base before further analysis. Gene network analysis was carried out using Ingenuity Pathways Analysis software (Ingenuity Systems Inc., CA, USA). Primary microarray data is available in NCBI's Gene Expression Omnibus public database (microarray platform, GPL6884; microarray data, GSE13898).

### Validation of Selected Genes as Prognostic Biomarkers Using Real-Time RT-PCR

Total RNA was extracted from the FFPE sections following the manufacturer's instruction manual (RecoverAll^TM^ Total Nucleic Acid Isolation; Ambion Inc., TX, USA). Selected genes (SPP1, SPARC, MMP1, TWIST1, CSPG2, SOX21, DKK3, CD93, AKR1B10, and LUM) were assayed by using real-time qRT-PCR with Taqman primers specific to each gene (Applied Biosystems, CA, USA). Real-time RT-PCR amplification was performed using the StepOne^TM^ and StepOnePlus^TM^ Real-Time PCR System (Applied Biosystems., CA, USA). Cycling conditions were 45°C for 10 minutes and 95°C for 10 minutes, followed by 40 cycles of 97°C for 0.02 minutes and 60°C for 0.30 minutes. Relative amounts of mRNA were calculated from the threshold cycle (C_T_) number using expression of cyclophilin A (PPIA) as an endogenous control. All experiments were duplicated and the values averaged.

### Statistical Methods

To select genes that are differentially expressed in two groups of tissues, we used the class comparison tool in BRB ArrayTools (v 3.6; Biometrics Research Branch, National Cancer Institute, MD, USA) to perform multiple comparisons of t-statistics with estimation of false discovery rate (FDR). During statistical analysis, one of duplicated experiments was removed to avoid redundant presentation of same sample. Cluster analysis was performed using the software programs Cluster and Treeview [16]. Associations between selected genes and prognosis were estimated by applying Kaplan-Meier plotting and the log-rank test. All statistical analyses were two-sided and done at a P<0.05 significance level.

## Results

### Distinct Subtypes of EAC Are Strongly Associated with Prognosis

We characterized gene expression profiles in 75 EAC and 28 NE tissue samples from 64 patients. **Table 1** lists the clinicopathological and demographic characteristics of the patients. To estimate the variance of gene expression of different biopsies from the same patients, gene expression data were also collected from two different biopsies of a single patient in 11 randomly selected patients.

**Figure 1:**
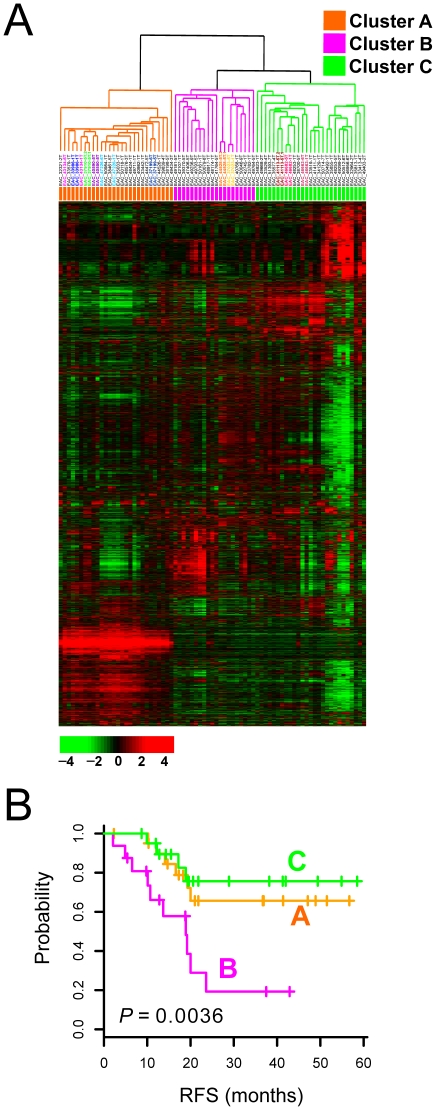
Hierarchical clustering analysis. (A) Hierarchical clustering of genes from 75 EAC tissues. Genes with an expression ratio that was at least twofold different relative to reference in at least 8 tissues were selected for hierarchical analysis (6,802 gene features). The data are presented in matrix format, with rows representing the individual gene and columns representing each tissue. Each cell in the matrix represents the expression level of a gene feature in an individual tissue. Red and green reflect high and low expression levels, respectively, as indicated in the scale bar (log 2 transformed scale). Duplicated biopsies from the same patients were highlighted in colors in dendrogram. (B) Kaplan-Meier plot of disease-free survival of EAC patients grouped on the basis of gene expression profiling.

An unsupervised hierarchical clustering analysis based on Pearson correlation coefficients was applied to all tissues from EAC patients on the basis of similarity in the expression pattern over all genes. As expected, EAC tissues were well separated from most of the NE tissues, clearly indicating that global gene expression fully reflects pathological and biological differences between NE and EAC (**Figure S1**). Interestingly, clustering analysis performed with only EAC tissues revealed three distinct subgroups with clear differences in overall gene expression patterns (**Figure 1A**). As measured in duplicated experiments with different biopsies from the same patient, the reproducibility of the data is extremely high. All duplicated experiments resided in nearest neighbors after clustering, strongly suggesting that the variance among experiments and biopsies was negligible. Therefore, most of the difference in gene expression largely reflected biological, as well as clinical, differences among the EAC cases.

Having three distinctive subclasses of EAC that may reflect clinical heterogeneity, we next examined the association between the clusters and clinical data. Kaplan-Meier survival curves indicated poorer prognosis of patients in cluster B (**Figure 1B**). Recurrence free survival (RFS) was significantly worse in cluster B patients than those in clusters A and C (*P* = 0.0036, by log-rank test). In addition, the mean overall survival (OS) of cluster B patients was much shorter (<13 month) than that of the rest of the patients. Thus, the molecular differences between the three subclasses of EAC that we identified were well associated with a remarkable difference in the clinical outcomes of these patients.

### Gene Expression Signature Is Strongly Associated with Prognosis

**Figure 2:**
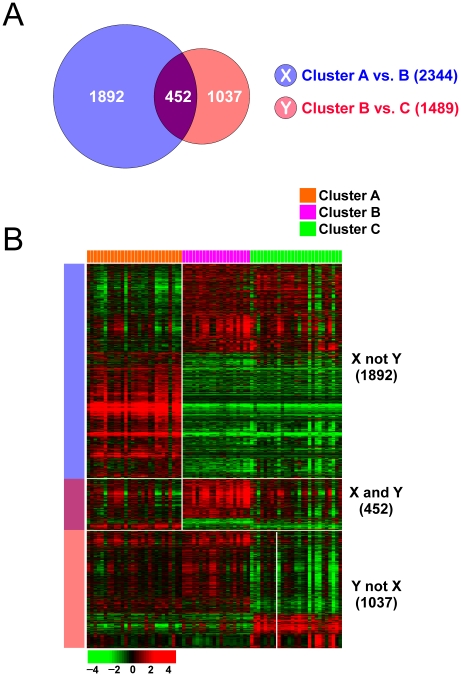
Cross comparison of gene lists from two independent statistical tests. (A) Venn Diagram of genes differentially expressed. The blue circle (gene list X) represents genes differentially expressed between cluster A and B. The red circle (gene list Y) represents genes differentially expressed between cluster B and C. Four-hundred-fifty-two genes were shared by the two gene lists. We applied a cut-off P-value of less than 0.002 to retain genes whose expression is significantly different between the two groups of tissues examined. (B) Heat map of gene expression patterns. Blue and pink bars on the left side of the heat map represent each selected genes. Colored bars at the top of the heat map represent the tissues indicated. Expression of genes in the X not Y category was dramatically different between clusters A and B as well as between clusters A and C, but almost no differences were observed between clusters B and C, signifying a unique gene expression signature that distinguishes patients in cluster A from the rest of the patients.

Since the most striking feature of the unsupervised analysis of the expression profiles was the strong association with prognosis and the presence of three subgroups of EAC, we next applied statistical analysis methods to uncover the genes whose expression patterns are best associated with EAC subgroup clustering and prognosis. We first sought to find gene sets that are differentially expressed in the three EAC subgroups (cluster A, B, and C). We generated two different gene lists by applying two-sample t-tests (*P*<0.002). Genes were further selected to have 1.5-fold differences between the groups compared in the t-tests. Gene List X (2,344 gene features) represents the genes that were differentially expressed between clusters A and B, whereas gene List Y (1489 gene features) represents the genes that were differentially expressed between clusters B and C (**Figure 2A**). When two gene lists were compared, 3 different sets were observed: X not Y (1,892), X and Y (452), and Y not X (1,037). Genes in the X and Y category displayed specific gene expression patterns relatively enriched in cluster B patients. Because patients in cluster B showed poorer prognosis, we further investigated gene expression data in the X and Y gene list (**Figure 2B**).

**Figure 3:**
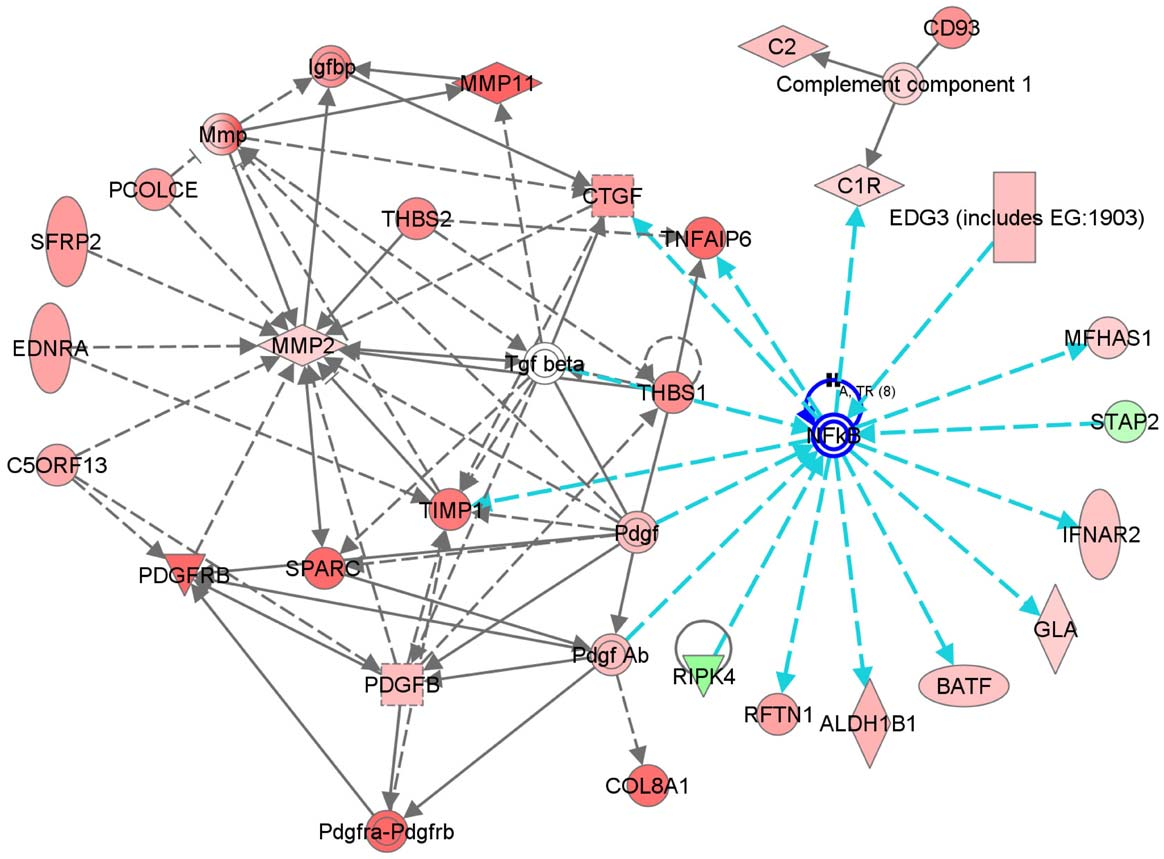
Gene networks from IngenuityTM Pathway Analysis. Global networks of inter-connection among genes and expression patterns of genes in network #1 in Appendix Table 1. Red and green colors in each shape indicate up- or down-regulation of expression in cluster B when compared with cluster A and C. Genes in gray color are not in the list but associated with the regulated genes. Each line and arrow represents functional and physical interaction and direction of regulation demonstrated in the literature. Genes inter-connected with NF-kB are highlighted in blue lines.

Having found a gene expression pattern well reflecting prognosis of EAC patients, we next tried to uncover gene networks that might be enriched in these genes. Gene network analysis using Ingenuity^TM^ Pathway Analysis (IPA) (Ingenuity Systems, CA, USA) was applied to the genes and their expression patterns. This analysis revealed a series of putative networks, of which the 20 with the highest scores are listed in **Table S1**. For example, functional connectivity of the top network (network #1) revealed a strong over-representation of NF-kB (**Figure 3**). Although the expression of NF-kB was not altered, the expression levels of many downstream target genes of NF-kB were up-modulated in patients in cluster B, strongly indicating that transcriptional activity of NF-kB is high in cluster B and might be responsible for the poorer prognosis of these patients.

### Exploration of Candidate Biomarkers

Regardless of strong association of the expression signature with prognosis, the large number of genes in the signature would hamper its clinical usefulness. To overcome this limitation, we next tried to identify a small number of genes whose expression patterns can reliably predict OS or RFS in EAC patients. Out of the 452 genes previously identified, we first selected the genes whose expression levels significantly differed in magnitude (≥4-fold) between patients in cluster B and those in clusters A and C. In order to minimize over-representation of particular gene networks or pathways in selected prognostic markers, we limited to two the maximum number of genes per predicted gene network (**Table S1**). Ten genes met these criteria, namely *AKR1B10, CD93, CSPG2, DKK3, LUM, MMP1, SOX21, SPP1, SPARC,* and *TWIST1*. Using hazard ratios from the univariate Cox regression analysis as indicators of survival, *AKR1B10* and *SOX21* are protective genes (associated with a hazard ratio of less than 1) and the others are risk genes (associated with a hazard ratio of more than1).

**Figure 4:**
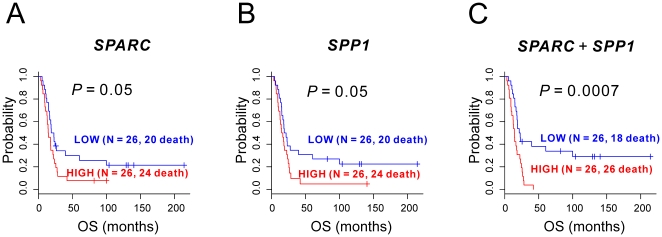
Kaplan-Meier plots of overall survival for EAC patients. (A) Overall survival by *SPARC* status in 52 patients. (B) Overall survival by *SPP1* status in 52 patients. (C) Overall survival by *SPARC*+*SPP1* in 52 patients.

Considering the 10 selected genes as representative prognostic molecular markers, we tested whether expression of the genes or their subsets could predict the duration of survival in an independent cohort. Before applying qRT-PCR in an independent cohort, we assessed the reliability of gene expression measurements in our microarray study by comparing them with those obtained using qRT-PCR in replicate samples. We isolated total RNA from FFPE tissues from 52 EAC patients and applied qRT-PCR with the use of specific TaqMan^TM^ probes and primer sets to the total RNA in order to measure gene expression. We first assessed the prognostic relevance of expression by applying Kaplan-Meier plotting and the log-rank test after dichotomizing patients. The median expression level of each gene was chosen as the cut-off value to ensure equal numbers of patients in poor and better prognosis groups. Expression of *SPARC* and *SPP1* was significantly associated (P = 0.05, by log-rank test) with OS of EAC patients in the validation cohort (**Figure 4A & B**). We tested whether combined expression patterns of two genes can improve the significance of association. When patients were dichotomized by averaged expression values of *SPARC* and *SPP1*, the association between the two genes and OS was highly significant (P = 0.0007, by log-rank test), strongly suggesting that the prognostic association of the two genes in EAC is synergistic (**Figure 4C**). However, prognostic association of the rest of genes was not significant (data not shown).

### Prognostic Utility of Gene Expression Signature in EAC

To evaluate the prognostic relevance of our newly discovered gene expression signatures, we applied univariate and multivariate analyses of the signatures using known clinical and pathological risk factors for EAC progression. In agreement with previous reports [2], [8], we found several tumor characteristics associated with OS using univariate Cox proportional hazards analysis (**Table 2**). In addition, multivariate analyses that included all relevant pathological variables revealed that gene signature was independent prognostic markers for OS of EAC patients. Therefore, our findings suggest that our gene signature retains its prognostic relevance, even after the “classical” pathological prognostic features have been taken into account. Moreover, the gene expression signature was revealed independently of the clinicopathological features of EAC tumors, indicating that the potential clinical utility of the signature might come from a better mechanistic understanding of EAC progression.

**Table 2:** Univariate and multivariate Cox proportional hazard regression analyses for overall survival in validation cohort.

	Univariate		Multivariate	
	Hazard Ratio (95% CI)	*P*-value	Hazard Ratio (95% CI)	*P*-value
**Two gene signature** **(SPARC & SPP1)**	2.87 (1.51–5.44)	0.0013	2.29 (1.162–4.68)	0.024
** pT** ** (1,2,3,4)**	1.70 (1.01–2.84)	0.045	0.89 (0.16–5.03)	0.900
** pN** ** (0–3)**	4.36 (1.77–10.7)	0.0014	3.01 (0.90–10.12)	0.075
** pM** ** (0–1)**	1.68 (0.71–4.02)	0.240	1.28 (0.14–11.73)	0.83
** Age** ** (>60)**	0.99 (0.55–1.79)	0.969	1.25 (0.62–2.53)	0.53
**Pathological Stage** # **(1,2,3,4)**	1.69 (1.15–2.49)	0.0081	1.11 (0.15–8.27)	0.92
** BE** ** (Y or N)**	0.94 (0.48–1.83)	0.852	1.42 (0.63–3.19)	0.39
** Gender** ** (M, F)**	1.55 (0.48–5.01)	0.468	1.50 (0.41–5.53)	0.54

# Pathological stages of tumor are re-assessed with after surgical resection of tumors.

## Discussion

Previous genome-wide studies on various cancers strongly support the notion that biological difference reflected in gene expression profiles of tumors may dictate the prognosis of cancer patients [9]–[13]. In the present study, we applied systems-level characterization of EAC transcriptome to address molecular heterogeneity of EAC and to extract a gene expression signature that can subdivide EAC patients to homogeneous groups with significant clinical and biological difference. In an attempt to address the molecular heterogeneity of EAC, we first applied unsupervised analysis of gene expression data, which revealed three subgroups of EAC. Significant association of the three subgroups with prognosis and distinct gene expression patterns within each subgroup led us to hypothesize that the unique gene expression patterns of each subgroup may reflect biological as well as clinical heterogeneity of EAC. For example, many of the selected genes whose expression patterns are associated with cluster B, the worst prognosis subgroup, are well known to be involved in metastasis/invasion (i.e., *SPP1, MMP1, MMP2, MMP3, TIMP1, CDH11,* and *TWIST1*) and proliferation (i.e., *CDK4* and *MCM2*). An intriguing feature among our 452 potential prognostic gene expression signatures is that around 80% of the genes showed a relatively high level of expression in cluster B patients when compared with the rest of the patients, indicating that gain, rather than loss, of gene activity may have more influence on the prognosis of EAC.

However, application of gene expression profiles to clinical practice is very challenging due largely to the difficulty to get fresh-frozen tissues from patients for microarray experiments and the complexity of data analysis with large number of genes. Therefore, we sought to develop methods that use quantitative real-time RT-PCR and RNA from easily accessible paraffin embedded specimen from patients. The robustness of the prognostic gene expression signature was validated in an independent cohort using the reduced gene set. Out of 10 prognostic markers tested, two genes (*SPARC* and *SPP1*) were significantly associated when gene expression patterns were measured by qRT-PCR in the independent cohort. SPARC is a matricellular protein that modulates cell adhesion and growth and modulates cell-matrix interactions by binding to the extracellular matrix [17]. High levels of SPARC are often associated with metastastic tumors [18]–[20]. In fact, *SPARC* has been proposed as a diagnostic marker of invasive meningiomas [21]. The expression of *SPARC* is correlated significantly with *MMP2* mRNA expression in esophageal tumor tissue specimens, and high *SPARC* expression was found to be correlated significantly with lymph node metastasis and poor patient prognosis [22]. SPP1, a secreted glycoprotein, also regulates cell adhesion and is well associated with metastasis in various cancers [23], [24]. In prostate cancer, elevated plasma SPP1 levels have been correlated with bone metastasis and poorer survival [25]. Thus, our findings concur with previous reports of these genes in other cancers.

Our data indicate that the prognostic gene expression signatures are present at the time of diagnosis. Therefore, the use of gene expression profiling promises to improve the molecular classification of EAC patients by adding to the existing classifications. Since our current method only use a very small amount of paraffin embedded tissues that are routinely acquired during diagnosis, we could potentially identify EAC patients with higher risk even before starting the treatment. Although our two-gene signature is examined in two independent cohort groups, robustness of signature should be further validated in larger cohort. Prospective multi-center studies would be ideal. Molecular stratification of EAC patients into homogeneous subgroups may improve the application of currently available treatments and provide opportunities for the development of new treatment modalities.

## Supporting Information

Figure S1 **Hierarchical clustering analysis of gene expression data from esophageal tissues.** Hierarchical clustering was applied to gene expression data from 75 EAC and 28 non-tumor esophageal tissues. Genes with an expression level that has at least 2-fold difference relative to median value across tissues in at least 18 tissues were selected for hierarchical clustering analysis (3,296 gene features). The data are presented in matrix format in which rows represent individual gene and columns represent each tissue. Each cell in the matrix represents the expression level of a gene feature in an individual tissue. The red and green color in cells reflect relative high and low expression levels respectively as indicated in the scale bar (log2 transformed scale).
(PDF)

Table S1 **Top 20 list of gene networks from IngenuityTM Pathway Analysis.**
(PDF)
