# Supplementary material for: Prognostic Biomarkers for Esophageal Adenocarcinoma Identified by Analysis of Tumor Transcriptome
Source: PLoS One. 2010 Nov 30;5(11):e15074. doi: 10.1371/journal.pone.0015074 (PMC2994829; doi:10.1371/journal.pone.0015074)
Supplement: Figure S1 — Hierarchical clustering analysis of gene expression data from esophageal tissues. Hierarchical clustering was applied to gene expression data from 75 EAC and 28 non-tumor esophageal tissues. Genes with an expression level that has at least 2-fold difference relative to median value across tissues in at least 18 tissues were selected for hierarchical clustering analysis (3,296 gene features). The data are presented in matrix format in which rows represent individual gene and columns represent each tissue. Each cell in the matrix represents the expression level of a gene feature in an individual tissue. The red and green color in cells reflect relative high and low expression levels respectively as indicated in the scale bar (log2 transformed scale). (PDF) [file pone.0015074.s001.pdf]

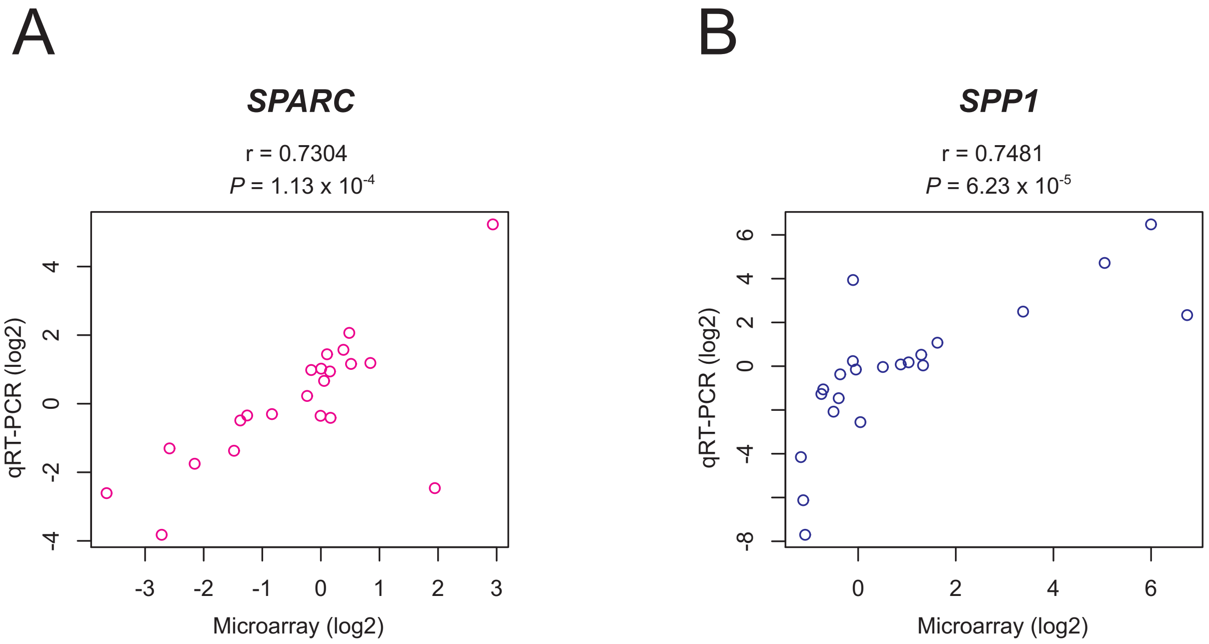

Figure S1 Comparison of *SPARC* and *SPP1* expression data obtained from real-time RT-PCR and microarray experiments.

Gene expression in RT-PCR experiments was normalized to the expression level of *PPIA* (cyclophilin A) in the sample and centralized by subtracting median expression value across samples. Gene expression data from microarray were also centralized by subtracting median expression value across samples.

(A) Scatter plots of normalized *SPARC* expression levels between two experiments from 24 EAC tissues. Pearson correlation coefficient between two experiments and significance of correlation coefficient is indicated at the top of plots.

(B) Scatter plots of normalized *SPP1* expression levels between two experiments from 24 EAC tissues.
