# Supplementary material for: Prognostic Biomarkers for Esophageal Adenocarcinoma Identified by Analysis of Tumor Transcriptome
Source: PLoS One. 2010 Nov 30;5(11):e15074. doi: 10.1371/journal.pone.0015074 (PMC2994829; doi:10.1371/journal.pone.0015074)
Supplement: Table S1 — Top 20 list of gene networks from IngenuityTM Pathway Analysis. (PDF) [file pone.0015074.s003.pdf]

Table S1 Top 20 list of gene networks from Ingenuity™ Pathway Analysis

	Molecules in Network	Score
1	↑**ALDH1B1,** ↑**BATF,** ↑**C2,** ↑**C1R,** ↑**C5ORF13,** ↑**CD93,** ↑**COL8A1,** Complement component 1, ↑**CTGF,** ↑**EDG3 (includes EG:1903),** ↑**EDNRA,** ↑**GLA,** ↑**IFNAR2,** Igfbp, ↑**MFHAS1,** Mmp, ↑**MMP2,** ↑**MMP11,** NFkB, ↑**PCOLCE,** Pdgf, Pdgf Ab, ↑**PDGFB,** Pdgfra-Pdgfrb, ↑**PDGFRB,** ↑**RFTN1,** ↓**RIPK4,** ↑**SFRP2,** ↑**SPARC,** ↓**STAP2,** Tgf beta, ↑**THBS1,** ↑**THBS2,** ↑**TIMP1,** ↑**TNFAIP6**	43
2	Akt, ↑**APLN,** ↑**CCT2,** ↑**CDC7,** ↑**CDC25B,** ↑**CDK4,** ↑**CHEK1,** ↑**CLEC11A,** ↑**COL18A1,** ↑**COL4A1,** ↑**COL4A2,** Cyclin A, Cyclin B, Cyclin E, E2f, ↑**EXOSC8,** ↑**FBLN2,** ↑**FHOD1,** Hsp90, ↑**HSP90AB1,** ↑**HYOU1,** ↑**IGFBP7,** ↑**ITGA5,** ↑**MCAM,** ↑**MCM2,** Mek, ↑**NID2,** ↑**NRP1,** ↑**PPT1,** Rb, ↑**SPOCK1,** ↑**SULF1,** ↑**THY1,** Vegf, ↑**WISP1**	41
3	↑**AEBP1,** ↑**ANGPT2,** ↑**ANTXR1*,** ↑**BUB1B,** Cbp/p300, ↑**CEBPB,** ↑**COL1A1,** ↑**CXCL2,** ↑**ENO2,** ERK1/2, ↑**FAP,** ↑**FCGR2A,** Fgfr, ↑**FSTL1,** IL1, ↑**IL6,** ↑**IL11,** ↑**IL24,** ↑**ITGA11,** JAK, ↑**LIF,** Mek1/2, ↑**OIP5,** p70 S6k, PI3K, ↑**PTP4A3,** ↑**RIPK2,** ↑**SNX10,** SOCS, ↑**SOCS4,** ↑**SPP1,** STAT, Tnf receptor, ↑**TNFRSF11B,** ↑**UBE2C**	36
4	↑**ADCY3,** Adenylate Cyclase, ↓**DHRS9,** ↑**DKK3,** ↑**ELK1,** ↑**ENTPD1,** ↑**EPHB1,** ↑**ESM1,** ↑**FHL3,** ↑**FNDC1,** G alphai, G protein beta gamma, ↑**GRM8,** Integrin, Integrinα, Integrinβ, ↑**ITGA1,** ↑**ITGB1,** Jnk, ↑**LGALS1,** ↑**MAD2L2,** ↑**NES,** Nfat, P38 MAPK, ↑**PGM2L1,** PLC gamma, Rac, Ras, ↑**RGS4,** ↑**RGS16,** ↑**SEPT4,** Sod, ↑**TGM2,** ↑**VCAM1,** ↑**ZAK**	32
5	Actin, ↑**ACTN1,** ↑**ADFP,** Alkaline Phosphatase, Ap1, ↑**CALD1,** CaMKII, ↑**CDC42EP2,** Ck2, ↓**CLOCK,** Creb, F Actin, Fibrin, ↑**FKHL18,** ↑**GAS1,** ↓**GSN,** ↑**HNRPD,** ↑**HTRA1,** LDL, Mapk, ↑**MMP1,** ↑**MMP3,** Myosin, ↓**PCAF,** PDGF BB, ↑**PKD2 (includes EG:5311),** Rxr, ↑**SCARF2,** ↑**STC1,** ↑**STC2,** ↑**THRA,** ↑**TPM2,** Tropomyosin, ↑**TWIST1,** ↓**ZNF433**	30
6	↑**ABCD1,** ↑**AEBP1,** ↑**ASPN,** ↑**BCL6B,** BMP1, BUB1 (includes EG:699), C5, ↑**CDH11,** CENPE, CLDN5, ↑**CMTM3,** ↑**COL5A1,** ↑**COL5A2,** COL5A3, DUSP4, ↑**EDNRA,** ↑**FZD2,** GJA7, ↑**GREM1,** hydrogen peroxide, ↑**IGFBP7,** ITGB6, MAPK1, ↑**PLOD1,** ↑**PLOD2,** ↑**PRKRIR,** Procollagen-lysine 5-dioxygenase, ↑**RCN1,** ↓**SCNN1A,** ↑**SERPINH1,** ↑**SH2B3,** ↑**SPARC,** TGFB1, TJP1, ↓**TJP3**	30
7	↑**ADAMTS5,** CBLC, ↑**COL3A1,** ↓**CROT,** ↑**CTSK,** ↑**CYB5B,** CYP2D9, EGFR, GH1, growth factor receptor, ↑**HS2ST1,** Igfbp, IGFBP2, ↑**ISLR,** ↑**ITGA1,** ↑**LINGO1,** ↑**LYPLA3,** ↑**MICB,** ↑**MTHFD2,** ↑**NNMT,** ↓**OAS1,** ODC1, PAX3, PBX1, PDCD6IP, PTPN12, retinoic acid, ↑**SCML1,** ↑**SH3KBP1,** SRGN, ↑**STC1,** TF, TIA1, ↑**VCAN,** ↑**ZNF281**	26
8	5'-methylthioadenosine, ADCYAP1R1, ↑**AURKA,** CHFR, CIAA1, CIAA2, ↑**COL10A1,** COL11A2, ↑**COL1A2,** ↑**COL3A1,** COL5A3, ↑**DNMT1,** ↑**EDNRA,** ELP2, ↑**FZD2,** HELZ, Histone h3, ↑**HSD11B1,** Insulin, IRS, ↑**PARP1,** PDZK1, Pka, Pkc(s), PLC, ↑**PTPRE,** RNA polymerase II, ↑**SCARB1,** ↑**SERPINH1,** ↑**SMOC2,** ↑**SMYD3,** STAT5a/b, ↑**STX2,** ↑**TM4SF18,** ↓**VAMP8**	22
9	↑**C5ORF13,** ↓**CDKN2AIP,** ↑**CKLF,** ↑**COL1A1,** ↑**COL4A1,** ↑**CTGF,** ↑**DKFZP762E1312,** DNA-directed DNA polymerase, ELAVL1, ↑**ENO2,** Glutathione peroxidase, GPX1, ↑**GPX7,** HDAC3, ID2, ↑**KIAA1199,** ↑**KNTC1,** MAF, ↑**MFAP2,** MYB, NBN, NME1, NOTCH1, POLG, ↑**POLG2,** PRDM5, ↑**QPRT,** ↑**RAB15,** RUNX1, RXRB, SOD2, ↑**TFAM,** ↑**TMSL8,** TP53, VHL	22
10	↑**ALPK2,** butyric acid, ↑**CDH11,** CHI3L1, ↑**COL4A1,** ↑**COL5A2,** ↑**COL6A1,** ↑**COL6A2,** ↑**COL6A3,** COL6A1 MAPPED, ↑**DNTTIP1,** ELL, F7, FBN1, FIGF, FOS, ↑**FSTL1,** GAPDH (includes EG:2597), ↑**GAS1,** GEMIN4, ↑**KCNJ8,** KCNJ11, ↑**LEPRE1,** MYCN, ↑**NOLA1,** NR3C1, ↑**POFUT1,** ↑**PROCR,** ↑**PRRX1,** PTPRN, ↓**RNF141,** SIP1, SMARCD1, SMN1, WEE1	22
11	AMH, ATPase, ↑**CD300A,** CDKN1A, chondroitin sulfate B, ↑**COL1A1,** ↑**CROP,** ↑**CTSK,** DDR1, ELN, FGF7, FGF13, Fgfr, FGFR2, FOXM1, ↓**FXYD3,** GTF2I, HSPA5, ↑**HYOU1,** IL1R1, ↑**KIAA1274,** KRAS, ↑**LGALS1,** lipoxin A4, ↑**LUM,** ↑**MMP3,** ↑**NFE2L3,** PTPN11, ↑**RAD51C,** S100A4, ↓**SDC1,** SHP, ↑**SLN (includes EG:6588),** ↑**SPAG5,** ↑**VCAN**	20
12	↑**ANGPTL2,** ↑**CALU (includes EG:813),** ↑**COL3A1,** CREB1, CTNNB1, dihydrotestosterone, ETV1, Frizzled, ↑**HTRA1,** INHBB, ↑**KLK1 (includes EG:3816),** LRP6, MME, ↑**MMP1,** ↑**NRCAM,** OSM, PBSN, ↑**PPIL5,** ↑**PTPRG,** ↑**QPCT (includes EG:25797),** SERPINA3, SFTPB, ↑**SLC7A6,** ↑**SNAI1,** ↑**SPARC,** TCF4, ↑**TDO2,** TERT, TF, ↑**TNFRSF11B,** Wnt, ↑**WNT2,** WNT4, WNT10B, WNT5A	20
13	↑**ADAMTS4,** BMYO, ↑**DYSF,** ↑**FADS3,** FBLN1, FIGF, FLNB, GLB1, ↑**ITGA5,** ITGA9 (includes EG:3680), ↑**ITGB1,** LAMA1, LAMA2, LAMA3, ↑**LAMA4,** LAMA5, LAMA4 PREDICTED, ↑**LAMB1,** LAMB3, LAMB1-2, ↑**LAMC1,** LAMC2, ↑**LGALS1,** ↑**NID2,** PLG, ↑**SCHIP1,** SMARCA4, ↓**SPINK5,** sulfatides, ↑**TEAD2,** ↑**TEAD4,** TNNT2, ↑**TRIM37,** ↑**VCAN,** VGLL1	20
14	ADM2, AGRP, ↑**ANGPT2,** ↑**ATIC,** ↑**CALCRL,** CRMP1, cyclic AMP, CYP27B1, ↓**CYP2C18,** Dihydropyrimidinase, DLK1, ↑**DPYSL2,** ↑**DPYSL3,** DPYSL5, E2F1, ↑**ENO2,** ↓**ETFDH,** ↑**H2AFX,** HAND1, HAND2, ↑**HEY1,** ↑**HEYL,** IGF1, L-triiodothyronine, LEP, ↑**MDC1,** MYH6, NPPA, POU1F1, RAMP1, RAMP2, RAMP3, ↑**TRPV2,** ↓**TSPO,** ↑**ZNF22**	19
15	↑**AHSA1,** ↓**AK3L1,** ↑**ANTXR2,** ↑**ATAD3A,** Ca2+, CANX, CAPZB, ↑**CHN1 (includes EG:1123),** ↑**CNTNAP1,** ↑**COL4A1,** DLG1, EEF1E1, ↑**ENPEP,** FCGRT, ↑**GPR124,** GRIN2A, ↑**H2AFZ,** HMGN2, HSP90AA1, LAMP1, ↑**MAP1A,** MYC, PEG3 (includes EG:5178), ↑**PODXL,** POLD1, ↓**RALGPS2,** RFX1, RFX3, SREBF1, ST14, TAC3, ↑**THBS2,** TP53, ↑**UBE2C,** WT1	19
16	ADORA2B, Caspase 3/7, CCL8, CCL18, CD163, ↑**CDCA5,** CDKN2A, ↑**CHTF18,** CLEC4E, ↓**CPEB3,** ↑**CST1,** CTSC, ↑**DCC1,** DERPC, ↑**FADS1,** GPNMB, ↑**IFI30,** IFNG, IL13, LAMP1, NFYB, NUP98, ↑**NUP107,** NUP133, NUP160, RFC4, RFX1, RFX5, ↑**SALL4,** ↑**SLC43A3,** TLR1, ↑**TMEM158,** ↑**TMEPAI,** ↑**USP13,** ↓**ZNF323**	17
17	beta-estradiol, ↑**CENPM,** ↑**CHST13,** ↑**CTGF,** estrone, FGF7, FOSL2, GFAP, ↑**GUCY1A3,** GUCY1B3, Igfbp, IGFBP4, ↑**ITGBL1,** LCN2, ↑**LYPD1,** Mmp, MMP7, ↑**MMP23A,** ↑**NKD2,** PLAT, ↑**PLEKHA4,** ↑**PLXDC1,** ↑**PRPS1,** PTTG1, ↓**SDCBP2,** SERPINA1, SERPINA3, SERPINB5, ↓**SH3RF2,** SOCS2, sulfotransferase, TFPI2, TGFA, TM4SF1, ZNF217	15
18	↑**ACOT9,** ↓**AKR1B10,** ARG1, ARNT2, BTNL2, CBFB, CCRN4L (includes EG:25819), ↑**COL11A1,** ↑**COL12A1,** ↑**COL15A1,** CYP27B1, ↑**DOCK11,** GBP2 (includes EG:14469)Δ, GBP2 (includes EG:2634), HSP90AA2, IFIT1L, IL4, IL19, IL33, IL10RA, LAMB3, ↑**MMD,** P4HB, PLP1, ↑**PPFIA1,** ↓**RAB27B,** SLC29A1, ↑**SYT11,** ↓**SYTL1,** Timp, TNF, ↑**TOMM34,** USP2, ↑**VASH1,** YWHAG	15
19	↑**CHSY1,** ↑**CSS3,** Glucuronyl-N-acetylgalactosaminylproteoglycan beta-1,4-N-acetylgalactosaminyltransferase, N-acetylgalactosaminyl-proteoglycan 3-beta-glucuronosyltransferase	3
20	↑**APOLD1,** leukotriene D4	2

The score is a numerical value used to rank networks according to how relevant they are to the genes in input dataset (406 genes). The score takes into account the number of genes in the network and the size of the network to approximate how relevant this network is to input gene list.
